# A Serious Game (Immunitates) About Immunization: Development and Validation Study

**DOI:** 10.2196/30738

**Published:** 2022-02-18

**Authors:** Isabela Dantas de Araujo Lima, Casandra Genoveva Rosales Martins Ponce de Leon, Laiane Medeiros Ribeiro, Izabel Cristina Rodrigues da Silva, Danielle Monteiro Vilela, Luciana Mara Monti Fonseca, Fernanda dos Santos Nogueira de Góes, Silvana Schwerz Funghetto

**Affiliations:** 1 Graduate Program in Health Sciences and Technologies Ceilândia College University of Brasília Brasília Brazil; 2 Ceilândia College University of Brasília Brasília Brazil; 3 Centro Universitário Claretiano Ribeirão Preto Brazil; 4 Ribeirão Preto College of Nursing University of São Paulo Ribeirão Preto Brazil; 5 MacEwan University Edmonton, AB Canada

**Keywords:** educational technology, immunization, nursing education, validation, methodological study, vaccination, public health, nursing students, teaching, education, support tool, continuing education

## Abstract

**Background:**

Vaccination is a fundamental part of all levels—local to worldwide—of public health, and it can be considered one of humanity's greatest achievements in the control and elimination of infectious diseases. Teaching immunization and vaccination can be monotonous and tiring. It is necessary to develop new approaches for teaching these themes in nursing school.

**Objective:**

We aimed to develop and validate a serious game about immunization and vaccination for Brazilian nursing students.

**Methods:**

We developed a quiz-type game, Immunitates, using design and educational theoretical models and Brazilian National Health Guidelines. The game’s heuristics and content were evaluated with 2 different instruments by a team of experts. A sample of nursing students evaluated the validity of the game’s heuristics only. We calculated the content validity index (CVI) for each evaluation.

**Results:**

The study included 49 experts and 15 nursing students. All evaluations demonstrated high internal consistency (Cronbach α≥.86). The game’s heuristics (experts: CVI 0.75-1.0; students: CVI 0.67-1.0) and the game’s contents demonstrated validity (experts: CVI 0.73-1.0). Participants identified some specific areas for improvement in the next version.

**Conclusions:**

The serious game appears to be valid. It is intended as a support tool for nursing students in the teaching–learning process and as a tool for continuing education for nurses.

## Introduction

Vaccination, a fundamental part of public health, is one of humanity's greatest achievements in the control and elimination of infectious diseases [1]. The benefits of vaccines have been proven; in the last few decades, as an example, smallpox was eradicated through immunization [2]. Vaccines have saved lives; reduced the incidence of polio by 99% in the world; and reduced diseases, disabilities resulting from diseases, and deaths caused by diphtheria, tetanus, and whooping cough [2].

Immunization of the population can decrease the transmission of infectious diseases, in turn decreasing hospitalizations, public expenditures on health care, and population mortality, which may increase the life expectancy of a population. Immunization will always be a necessary health action. Each year, 130 million babies are born [3], and they all have the right to receive protection against vaccine-preventable diseases.

In Brazil, it is necessary to abide by the recommended vaccination schedule [4] for children to be considered immunized. Thus, it is important that those responsible for a child are knowledgeable about the importance of vaccination—why the entire population must take the recommended vaccines—and that myths are debunked and explained.

Therefore, it is important that health professionals, especially nurses, have theoretical knowledge that offers a foundation and security to organize and promote health education. In this sense, nursing students must learn enough to provide health education to their future patients. Furthermore, in Brazil, nursing duties in primary care include administering vaccinations and managing everything related to them to ensure the safety of the immunobiological materials, and consequently, that of the patient [5].

In Brazil, the theoretical topics related to vaccination are mostly found in manuals and ordinances—technical texts that can be long, boring, and unappealing—resulting in dense and exhaustive reading for the student. We believe the use of a different methodology (serious games) in the teaching–learning process will help teachers to stimulate learning in the classroom and students to reinforce and review their knowledge on immunization. By using new methodologies, teaching can be more dynamic and the pattern of traditional vertical teaching, wherein the teacher demonstrates and the student repeats, can be broken [6]. Breaking this pattern of traditional education is important given the profile of the current generation of students—they are no longer just listeners but critical protagonists in the process of building knowledge [7,8].

The use of educational technologies in nursing has gradually increased over the years in Finland [9] and in other regions (Norway, other European countries, Asia, and Brazil [10]). To the best of our knowledge, there are no digital serious games related to vaccination in Brazil that are specifically for nursing students and nurses. We aimed to develop serious game about vaccine-preventable diseases and immunization and validate the game’s contents and heuristics.

## Methods

### Game Development

#### Framework

As a theoretical frame of reference for the creation and development of the game, we used the Elemental Tetrad (Story, Aesthetics, Mechanics, Technology) [11] and the first 3 levels (Remembering, Understanding, and Applying) of the Revised Bloom’s Taxonomy [12], as they have been commonly used in studies [13-16] that addressed the development of educational health technologies.

The contents of the game (a database with questions, answers, and feedback) were based on the Brazilian Ministry of Health's Manual of Rules and Procedures for Vaccination [17].

#### Story

**Figure 1:**
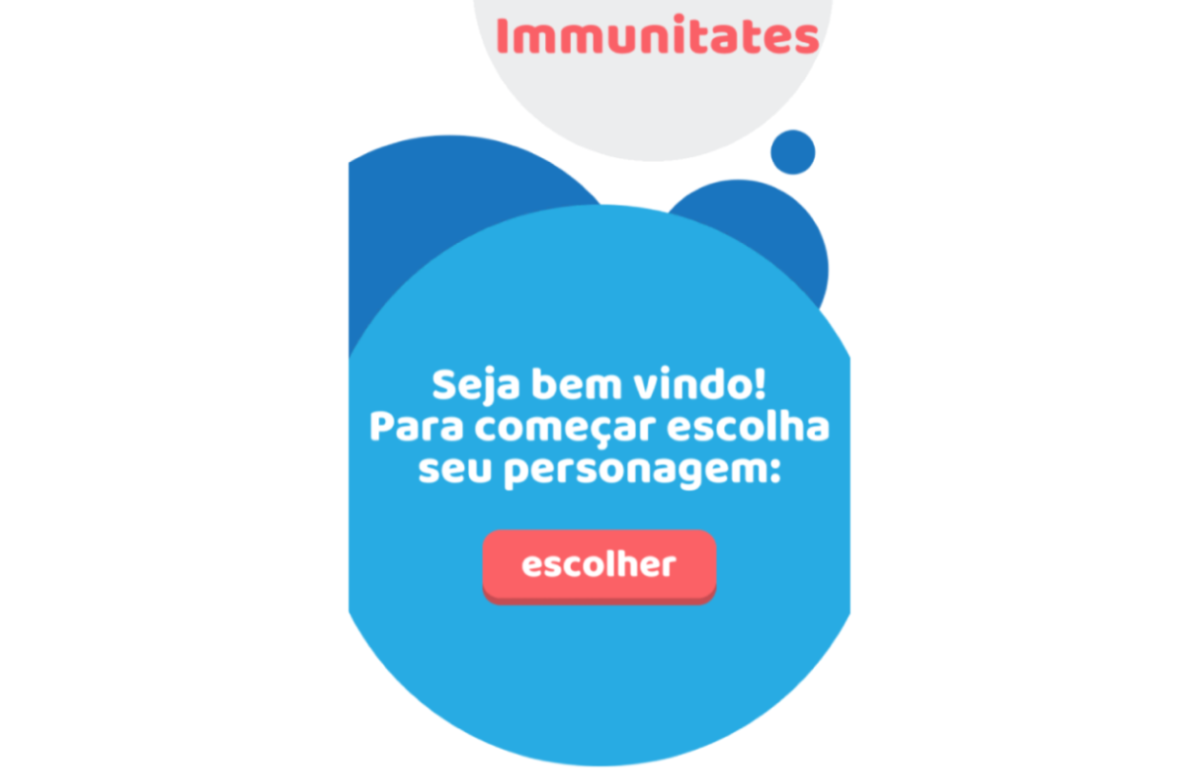
Welcome screen.

In the game *Immunitates* (Figure 1), the player assumes the role of nurse in the vaccination room and answers questions on immunization. When the players correctly answers a sufficient number of questions, their nurse avatar is promoted. The aim is to reach the last level of the game when the nurse reaches the role of Minister of Health.

Feedback on each question is immediate, thus maintaining the user's motivation and engagement in the game [18].

#### Aesthetics

**Figure 2:**
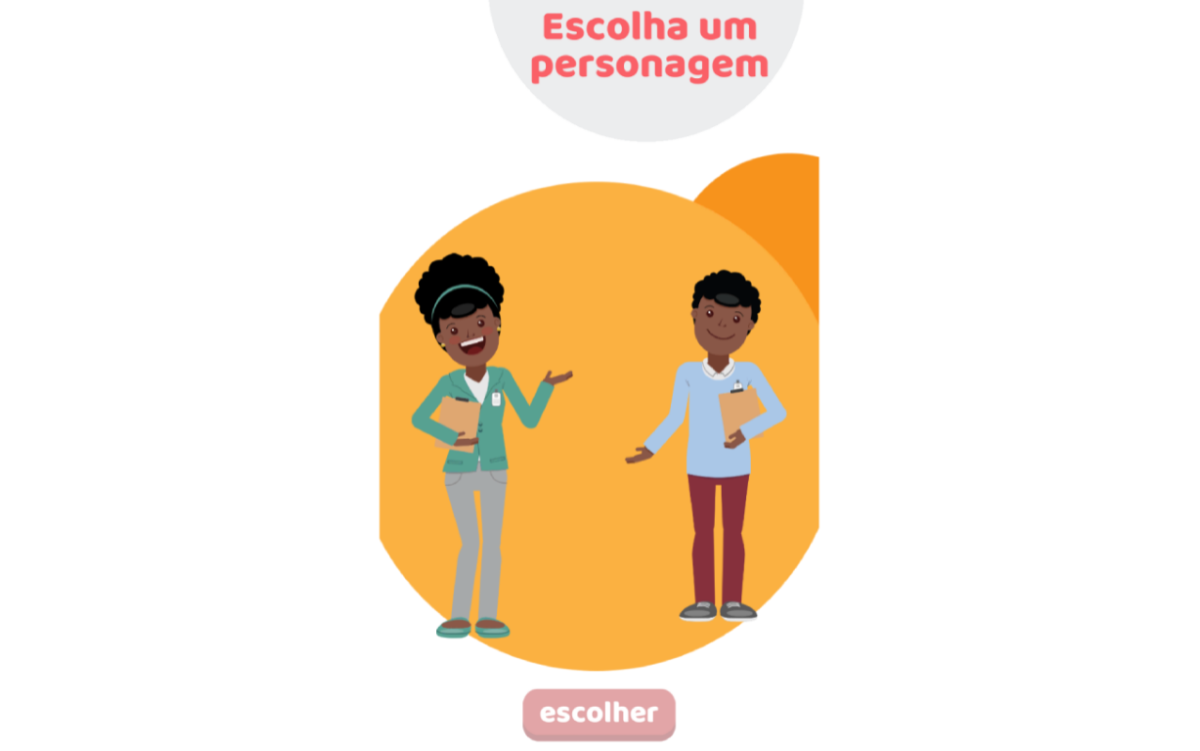
Avatar selection screen.

The player is asked to choose a female or male avatar (Figure 2), which changes job positions as the player advances to the next levels.

#### Mechanics

**Figure 3:**
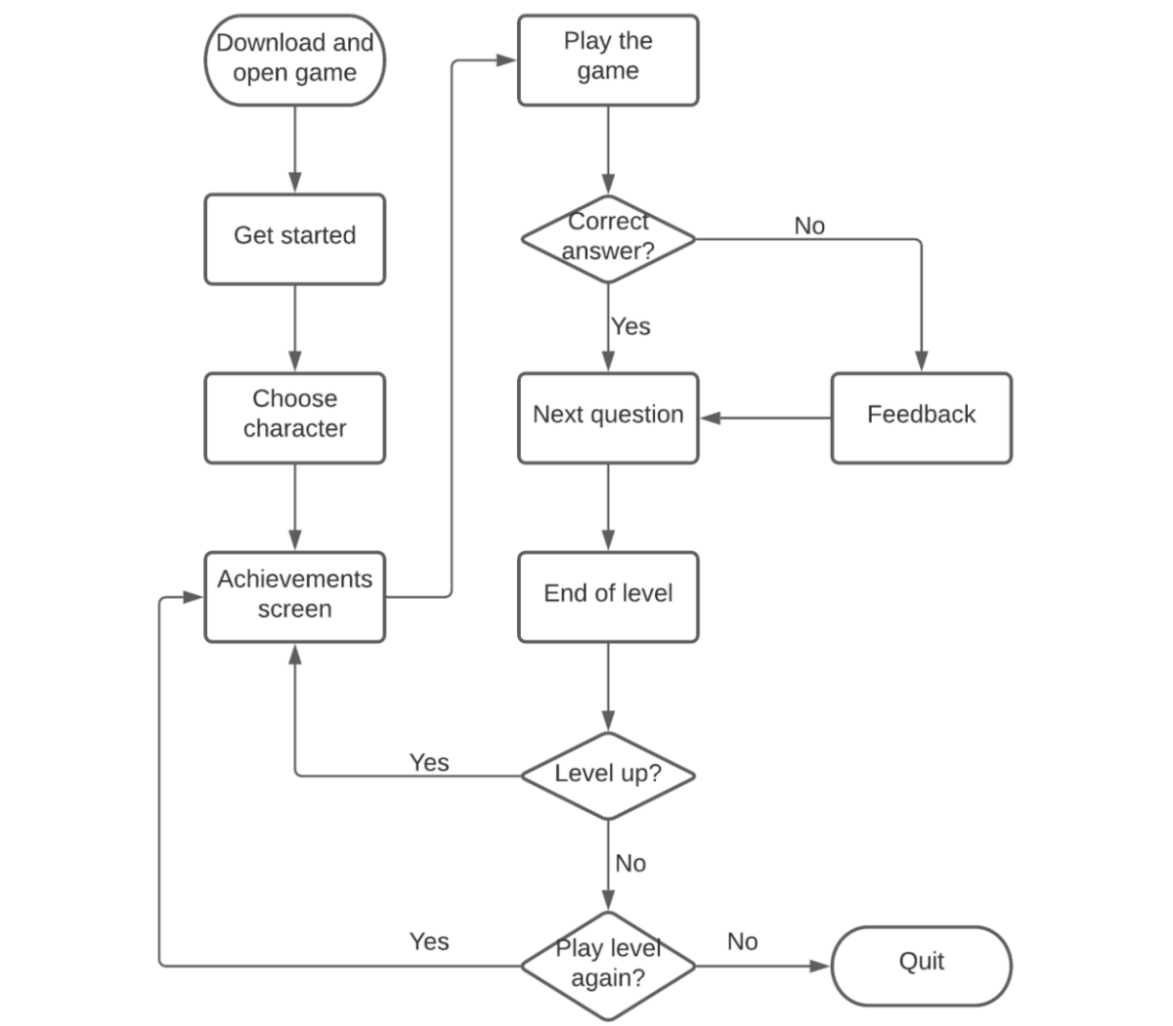
Immunitates flowchart.

**Figure 4:**
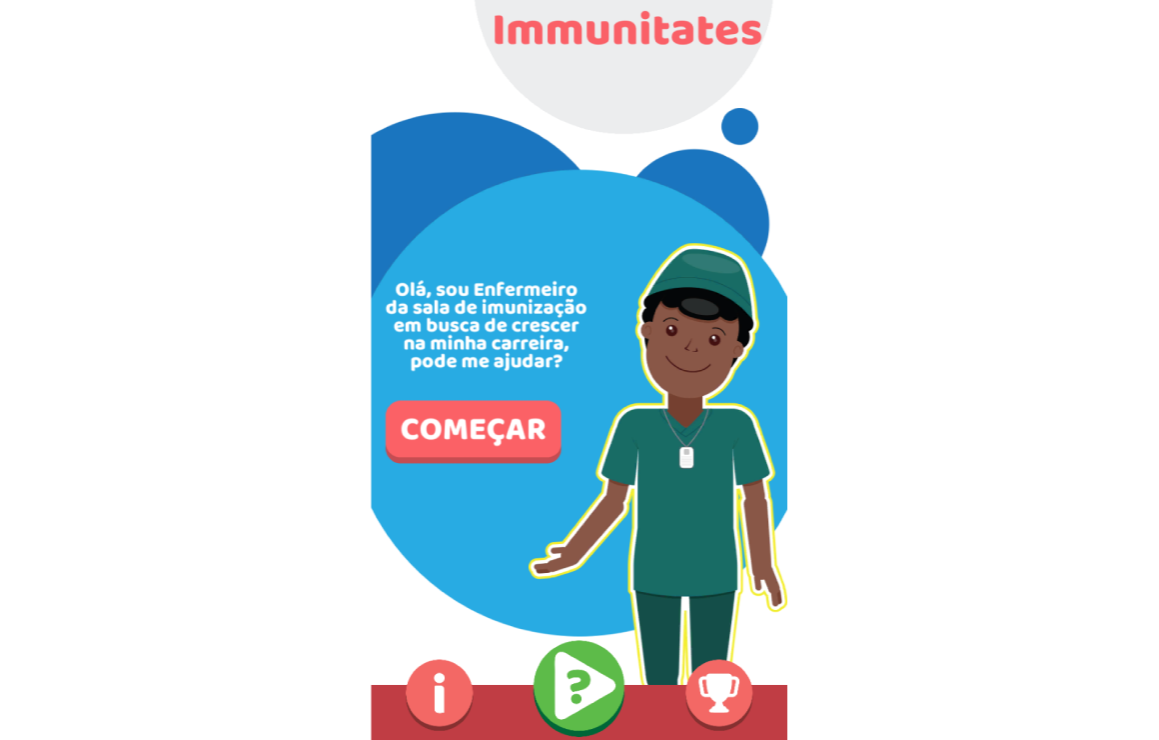
Screen explaining the game’s story.

**Figure 5:**
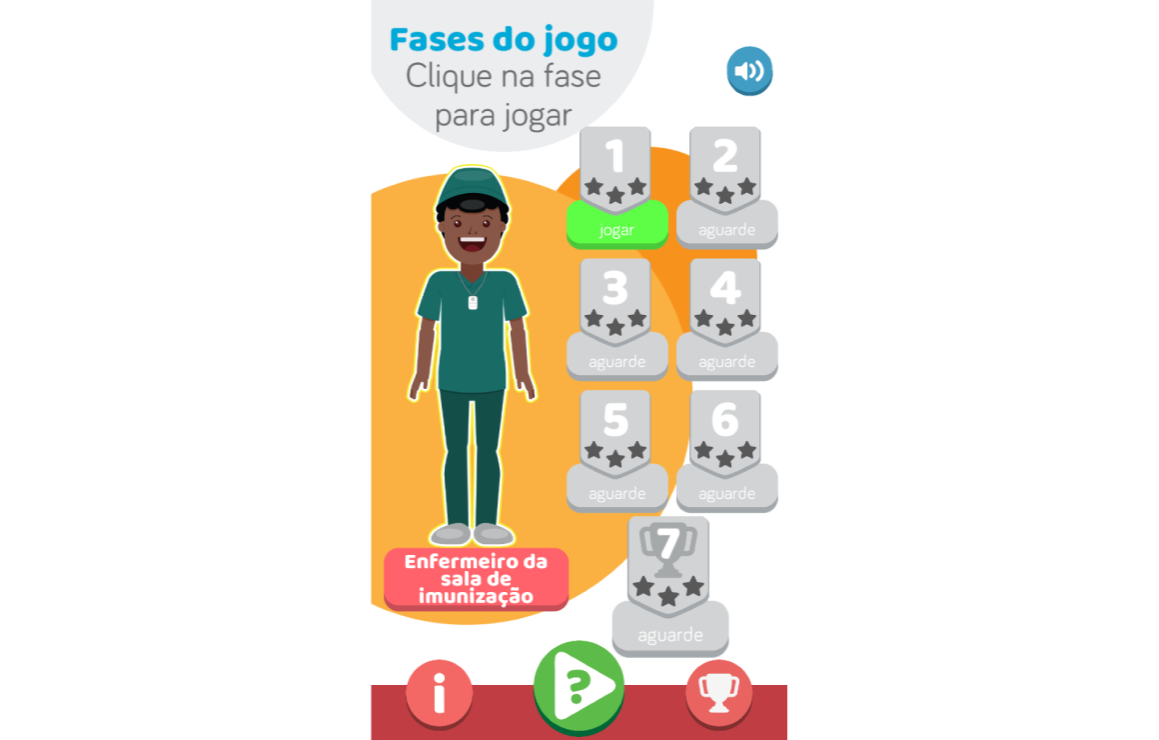
Achievements screen.

**Figure 6:**
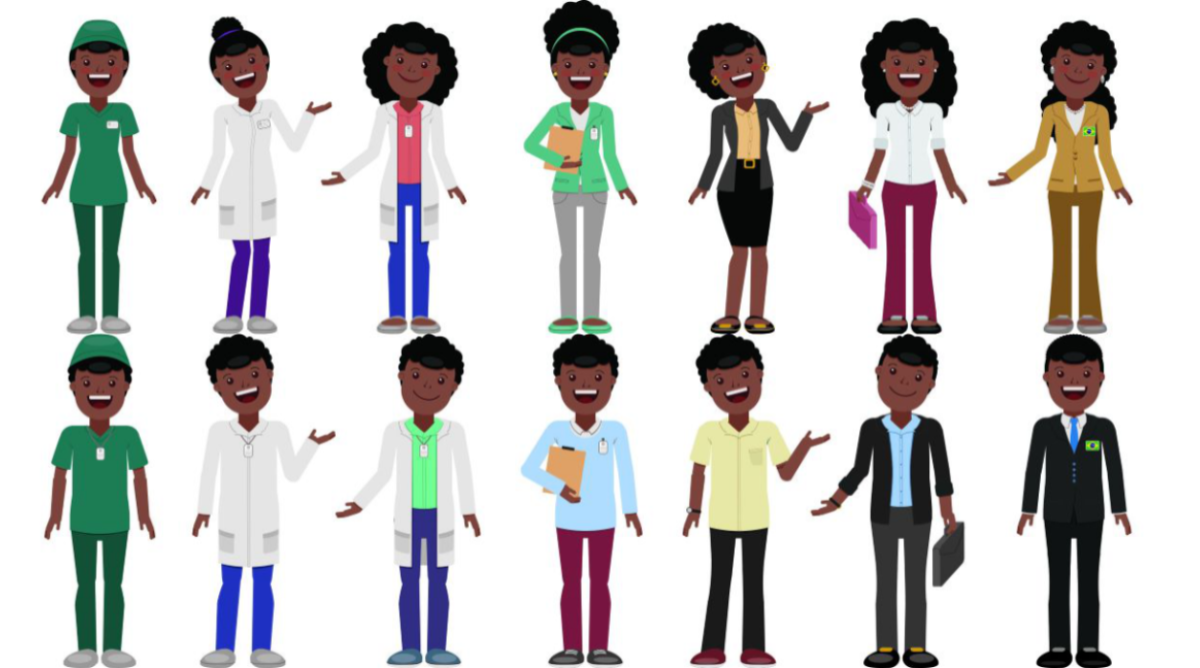
Female and male avatars.

The game has a quiz format, and players progress by levels (Figures 3-5), which are unlocked as the player answers questions correctly. The game has 7 levels, each corresponding to a job position that the nurse avatar achieves (Figure 6). Each level has a bank of questions, from which a specific number of questions are randomly chosen (Table 1). For example, level 1 has a bank of 5 questions, and each time the player accesses the level, 3 of the 5 questions are chosen. The player must correctly answer at least 1 question for the next level to be unlocked. If the user does not correctly answer the minimum number of questions, they fail the level but can immediately play it again.

**Table 1:** Game levels.

Level	Questions in database, n	Questions played, n	Correctly answered questions to progress to the next level, n
1 Immunization room nurse	5	3	1
2 Nurse manager of the Basic Health Unit	7	5	2
3 Director of the Basic Health Unit	9	6	3
4 Director of Health Surveillance	11	7	3
5 Health secretary	13	8	4
6 Director of the National Immunization Program	15	9	4
7 Health minister	20	10	5

**Figure 7:**
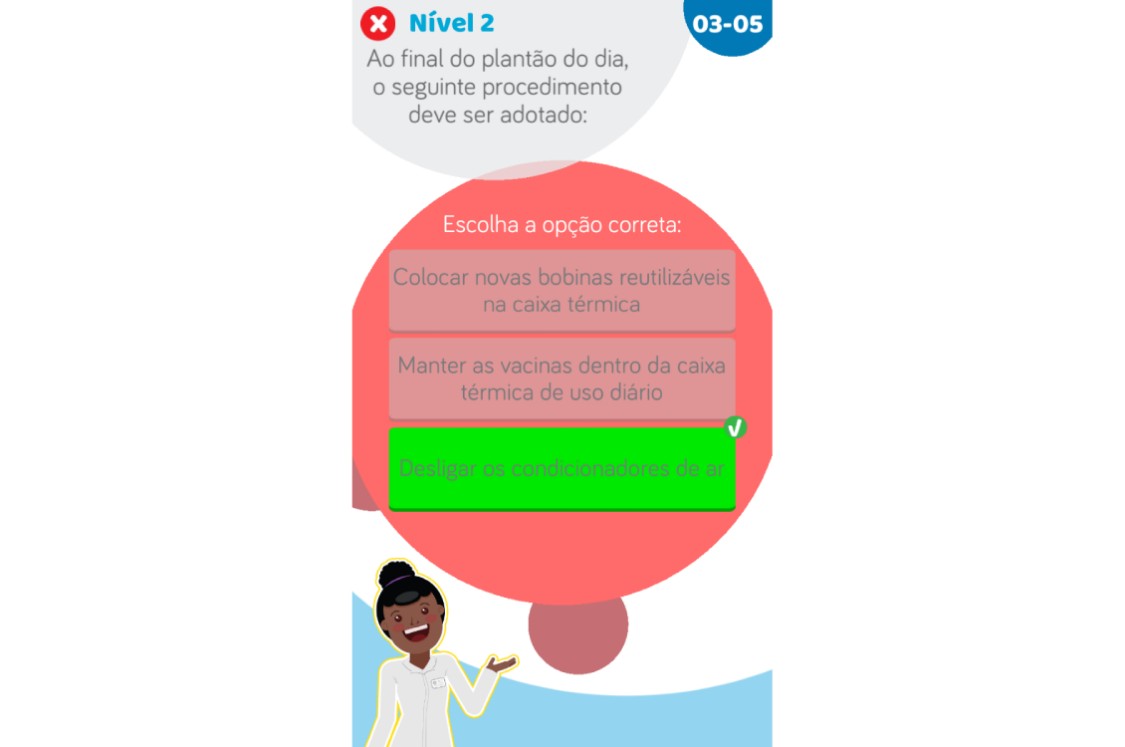
Correct answer screen.

**Figure 8:**
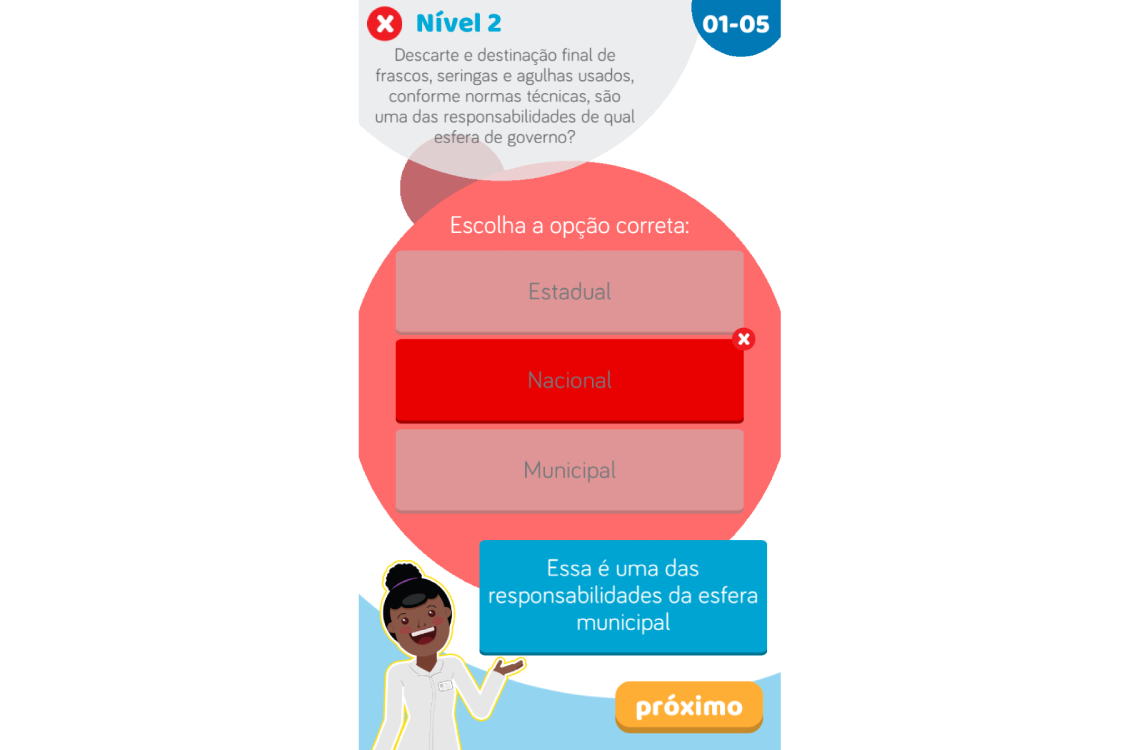
Incorrect answer screen with feedback.

The question database for the entire game consists of 80 questions. For each question, there are 3 options, with only 1 correct option (Figure 7), and text is automatically displayed to explain the correct answer, as automatic rationale feedback, if the player selects the incorrect option (Figure 8). Questions written with direct and simple wording and that require players to remember information were considered easy questions. Questions that require complex clinical reasoning were considered difficult and are presented later on in the game. For better visualization of the question and answer texts, questions could be up to 150 characters long, while each answer and feedback could be up to 120 characters.

Throughout the game, the player listens to a theme song (which can be muted on the achievements screen). The player also has the option to reset the game on the information screen (not pictured), this action restarts the game completely and all progress is lost.

#### Technology

The game was programmed in January 2019 using C++ and JavaScript for mobile phones with Android and iOS operating systems; the game is available in Portuguese for free from the Google Play Store and the Apple App Store.

### Game Validation

This study was undertaken with 2 different independent samples: (1) experts in nursing education technologies, nursing games, or immunization and (2) nursing students.

#### Experts

Inclusion criteria for experts were adapted from an existing model [19] that is widely used in Brazil to select experts for validation studies [20]. It was a mandatory criterion to have a Bachelor of Science degree in Nursing. An additional criterion was having specialized knowledge, clinical practice, or authorship in areas related to immunization, technology, or health education.

We actively searched 90 public nursing colleges in Brazil for nursing professors who had a history of researching nursing education technologies, nursing games, or immunization. We contacted 983 professors by email, inviting them to validate the game’s heuristics and content. The email had 2 different links: one to download the game, and the other to provide demographic information (age, gender, time working as a nurse, and additional degrees or certifications) to indicate that they accepted to be part of the study. Participants received a second email with links to heuristics and content evaluation forms.

#### Students

Inclusion criteria for students were being an undergraduate nursing student enrolled at University of Brasília Ceilândia College in the discipline *Comprehensive Care for Women and Children’s Health* at the time of data collection. In this discipline, students learn about the Brazilian vaccination calendar and immunization, and students were invited, during a class, to take part in this study. Students who agreed to participate were sent an email with 3 links: one to download the game, one to provide demographic information (age and gender), and a third to validate the game’s heuristics only.

### Ethics

The study was conducted in accordance with the Helsinki Declaration and Resolution 466/2012 [21] of the National Health Council (Brazil). The study was approved by the Research Ethics Committee of Ceilândia College, University of Brasília (CAAE 08595019.2.0000.8093).

### Evaluations

Game heuristics were evaluated by both experts and nursing students using *Avaliação Heurística para Jogos Educacionais Digitais* (AHJED, *Heuristic Evaluation for Digital Educational Games*) [22]), which has 8 dimensions—interface, playability, multimedia, artificial intelligence, game’s story, educational elements, contents, and educational agent—evaluated on a Likert scale (Multimedia Appendix 1).

Game contents were evaluated by experts using an instrument [23] with 3 dimension—objectives, structure and presentation, and relevance (Multimedia Appendix 2) and by omitting items about functionalities that were neither present nor intended to be present in the game.

Participants also had the option to leave comments about their experience playing the game and suggestions about what could be improved in the next version.

### Analysis

Statistical analysis was performed using SPSS software (version 22; IBM Corp).

The content validity index (CVI) is widely used in the field of health [24-26] and is used to assess the proportion of agreement for an item of an instrument [27]. The index was calculated in 2 ways: For the game heuristics evaluation, the number of *agree* and *strongly agree* responses were summed and divided by the total number of responses. For the game content evaluation, the number of *adequate* and *totally adequate* were summed and divided by the total number of responses. Items with CVI≥0.80 would remain as they are, whereas items with CVI<0.80 should be changed for the next version of the game.

We also calculated Cronbach α [28], a statistical measure that estimates the internal reliability of a questionnaire, for each evaluation (experts’ AHJED, students’ AHJED, and experts’ content). Cronbach α≥.70 was deemed acceptable [29].

## Results

Of those invited, 184 nursing professors provided demographic information in response to the first email, but only 49 completed the game validation and met inclusion criteria; therefore, 49 experts took part in this study. Of the 49 experts, 43 experts (88%) were female, and 6 (12%) experts were male. Experts ranged from 28 to 63 years old (mean 44.04 years old), with time working as a nurse ranging from 3 to 40 years. Of 48 students enrolled in the discipline, 15 took part in the study. Of the 15 students, 14 (93%) students were female, and 1 (7%) student was male. Students ranged from 20 to 28 years old (mean 22.13 years old).

Overall, the CVI ranged from 0.77 to 0.97. For heuristics, the CVI ranged from 0.75 to 1 in the expert group and 0.67 to 1 in the student group. Cronbach α was always greater than 0.86 (Table 2; Multimedia Appendix 3).

For content, the CVI ranged from 0.73 to 1, overall ranging from 0.88 to 0.93 (Table 3). Cronbach α was always greater than 0.89. Detailed results can be seen on the original tables in the supplementary files (Multimedia Appendix 4).

Participant comments were translated from Portuguese to English by the authors of this study. Not all participants chose to leave a comment. Both experts and students left suggestions related to the content, aesthetics, and their experiences playing the game (Table 4).

**Table 2:** Evaluation results for Immunitates heuristics.

Dimension	Experts (n=49)		Students (n=15)	
	Cronbach α	Content validity index	Cronbach α	Content validity index
Interface	.87	0.90	.88	0.93
Playability	.87	0.88	.88	0.94
Multimedia	.87	0.87	.88	0.97
Artificial intelligence	.87	0.90	.88	0.89
Game’s story	.86	0.90	.88	0.93
Educational elements	.86	0.90	.88	0.87
Contents	.87	0.83	.88	0.77
Educational agent	.87	0.86	.87	0.76

**Table 3:** Evaluation results for Immunitates content.

Dimension	Experts (n=49)		Students (n=15)	
	Cronbach α	Content validity index	Cronbach α	Content validity index
Objectives of the content	.90	0.88	—^a^	—
Structure and presentation of the content	.90	0.90	—	—
Relevance of the content	.89	0.93	—	—

^a^Only experts evaluated the content.

**Table 4:** Comments and suggestions from experts and nursing students.

Participant	Comment
Expert 2	“I suggest including more in-depth questions on the topic, to stimulate research and interest in the continuity of the game.”
Expert 4	“I believe that the feedback when we make mistakes should come with a suggestion for reading.”
Expert 7	“I suggest you remove the circles that are moving around, it takes your attention and makes it tiring.”
Expert 10	“Suggestion: enable a ‘learn more’ hyperlink; the player will be able to download the vaccination calendar, vaccination manuals and be redirected to the Ministry of Health webpage.”
Expert 15	“The font size for some questions is a little small. I suggest keeping the same size, if possible.”
Expert 25	“You should improve the options for questions and answers, making them shorter and easier to understand.”
Expert 27	“I felt the need for more complex questions, which would need clinical reasoning.”
Expert 28	“I suggest the themes to be worked on levels of complexity.”
Expert 49	“I really liked the game.”
Student 7	“The way the game approaches the subject is very good and helped me a lot.”
Student 11	“I believe the game should show a small screen with information on the topic when we answered a question wrong.”
Student 12	“Very good game.”
Student 15	“I wish there were more levels.”

## Discussion

The results present evidence of validity and suggest that the game can be used by both nurses and nursing students alike, as the game was tested and well accepted by them. We noticed that digital serious games about vaccination have up until now been developed for children [30,31].

Evaluations demonstrated high internal consistency (Cronbach α≥.86) [32]. Although most items within the evaluated heuristics were greater than 0.80, a few—level of difficulty (students: CVI 0.73), partition of the content (students: CVI 0.67), player’s performance (students: CVI 0.67), and aesthetics of the game (experts: CVI 0.75; students: CVI 0.67)—did not reach this stipulated number; these features will be reworked for the next version of the game based on suggestions:

I suggest including more in-depth questions on the topic, to stimulate research and interest in the continuity of the game.

Expert 2

I suggest you remove the circles that are moving around, it takes your attention and makes it tiring.

Expert 7

The font size for some questions is a little small. I suggest keeping the same size, if possible.

Expert 15

I wish there were more levels.

Student 15

For content, CVI≥0.88. Still, for some items in groups 1 and 2, CVI values were below expectations. Experts suggested that the content did not instigate change in behavior and attitude (CVI 0.73),

I felt the need for more complex questions, which would need clinical reasoning,

Expert 27

and that the messages were not clear or objective (CVI 0.78):

You should improve the options for questions and answers, making them shorter and easier to understand.

Expert 25

I suggest the themes to be worked on levels of complexity.

Expert 28

The content will also be reworked for the next version of the game.

The validation of educational technologies has been widely used in Brazil [23-25,33], and worldwide, in countries such as the United Kingdom [34], Canada [35], Italy [36], and Germany [37].

According to Korhonen [38], playability should not be the main objective of a game, although it is necessary for a positive experience, as good playability positively affects the user experience. Our results confirm this, since both experts (CVI 0.87) and students (CVI 0.88) indicated playability was acceptable, and expert and student participants commented on their good experience and how they liked the game:

Very good game.

Student 12

I really liked the game.

Expert 49

Content is perhaps the most important part of an educational tool. From participants’ suggestions, it was clear that both experts and students would have liked to have an external link that could provide theoretical support as a way to further their knowledge.

Expert 2 suggested including more in-depth questions to stimulate research and interest in the player, which corroborates the findings of a previous study [39], namely, that the difficulty of a game can affect player motivation. The game must have challenging elements but still leave the player confident enough to overcome them. Experts 27 and 28 also commented on the need for more complex questions that would need clinical reasoning, but expert 25 thought that the questions should be simpler, shorter, and easier to understand.

However, players may have different skills; what is classified as difficult, a player may perceive to be very easy or very difficult, which can lead players to feeling frustrated or even bored, which decreases their motivation and engagement with a game [40].

It is also true that technology-specific problems can arise when using educational technologies, such as issues downloading or installing and log-in, audio, and video-related problems. Students can find this method of teaching to be unengaging or they may simply not find the time to study using these new tools [41].

During the COVID-19 pandemic, when teachers and students worldwide had to stay at home, a new challenge arose in health care education. Somehow training in a field that is traditionally taught with a hands-on approach had to continue in alternative manners. During the first wave, educators began to utilize new educational technologies to continue teaching. The impact of these technologies in the field of health care education is unique because educators must continue training future professionals who will soon be working in-person at hospitals amid the pandemic, despite social distancing [42]. Although many educators have had to improvise and quickly learn how to use these new technologies during the COVID-19 pandemic, the use of technology in the teaching–learning process should be driven by pedagogical needs and goals and not by technological pressures [43].

When elements of gamification, such as points, achievements, and levels, are incorporated into the undergraduate teaching–learning process, there is a positive effect on student motivation and performance [44]. Motivation is the state in which the individual feels moved to do something. According to the Self-Determination Theory, motivation is separated into levels (from a little to a lot of motivation) and orientation (intrinsic and extrinsic motivation). In extrinsic motivation, the individual seeks reward or escapes punishment, whereas intrinsic motivation refers to pleasure to do or an inherent personal satisfaction [45]. Harandi [46] highlights that when students are motivated to learn, they are likely to be involved; when they are involved, they are more likely to achieve educational goals.

There were a few limitations in this study. The budget available for the construction of the game did not allow for all intended features to be included in this version. Suggestions made by the participants were documented so that, in the future, they can be implemented.

The number of students who participated was small, which can alter the results of the evaluation of educational technology. Students who had already completed the discipline *Comprehensive Care for Women and Children's Health* were not included, even though they already had the necessary knowledge to validate the game, because these students were engaged in course work outside the university, and we had difficulty contacting them.

In future studies, we intend to test the effectiveness of the game by comparing knowledge acquisition from class only compared with that from the combination of class and playing the game to determine if this game is useful in helping nursing students. We also intend to test graduate nurses’ satisfaction with the game and knowledge acquisition from the game to determine if it is useful to this target audience. With these results, we would be able to categorically affirm that the game is useful to both nurses and nursing students.

Immunitates presents evidence of validity, even though some areas of the game require improvement. Immunitates is designed to be a support tool for nursing students (ie, not to replace face-to-face class instruction) and to be a tool for continuing education for nurses.

## Appendix

Multimedia Appendix 1 Instrument for heuristic validation.

Multimedia Appendix 2 Instrument for content validation.

Multimedia Appendix 3 Heuristic validation results.

Multimedia Appendix 4 Content validation results.

## Abbreviations

AHJED: Avaliação Heurística para Jogos Educacionais Digitais (Heuristic Evaluation for Digital Educational Games)
CVI: content validity index
