# Supplementary material for: A Serious Game (Immunitates) About Immunization: Development and Validation Study
Source: JMIR Serious Games. 2022 Feb 18;10(1):e30738. doi: 10.2196/30738 (PMC8900905; doi:10.2196/30738)
Supplement: Multimedia Appendix 1 [file games_v10i1e30738_app1.docx]

Multimedia Appendix 1

Instrument for heuristic validation: AHJED (Avaliação Heurística para Jogos Educacionais Digitais) (in English: Heuristic Evaluation for Digital Educational Games) by Azevêdo, Rousy & Siebra.

| Dimension | Heuristic |
| --- | --- |
| Interface | The user knows their location and easily visualizes their status in the game |
|  | The user has free control over their actions in the game environment |
|  | The elements in the game are consistent and standardized |
|  | The elements of the game can prevent the user from accidentally performing an action |
|  | The elements of the game are suggestive enough to allow the user to play without having to resort to manuals and other types of help |
|  | The elements of the game allow the user to perform their tasks efficiently, that is, with as little effort as possible |
|  | The number of elements in the game is sufficient for the user to achieve their goals without confusing them |
|  | The game has elements that help the user to recognize, diagnose, and recover from the mistakes made |
| Playability | The game provides enough information for the user to start playing |
|  | The game's control keys follow conventional standards |
|  | Users can save games in different states throughout the game |
|  | Users who have successfully completed a game stage are rewarded |
|  | Game elements, for example, challenges, should not frustrate game users |
|  | The elements of the game give the user a sense of immersion, that is, it allows them to feel part of the environment, being able to identify and interact with objects in the scene |
| Multimedia | The user must be able to recognize in the multimedia elements of the game, what are the objectives contemplated by them |
|  | There is a correspondence between the multimedia elements used in the game and the learning contents contemplated in it |
|  | The combinations between the multimedia elements used in the game and presented to the user are consistent and representative |
|  | The quality of the multimedia elements used is sufficient for the user to understand their purpose (objective) |
|  | The multimedia elements of the game contribute to the presentation of the learning content, making it more attractive |
| Artificial intelligence | The game's artificial intelligence is consistent |
|  | The game's artificial intelligence is balanced with the player's skill |
|  | The game’s artificial intelligence has no flaws or inconsistencies |
| Game’s story | The player understands the story of the game clearly and consistently |
|  | The story promotes immersion |
|  | The game transports the player to a level of emotional involvement. The player is interested in the characters, because (1) they are like me; (2) they are interesting to me, (3) the characters develop as the game progresses |
| Educational elements | The user must be able to recognize in the elements of the game, what are the learning objectives contemplated by them |
|  | The game should allow its users to have greater autonomy of the learning process, (re) configuring their activities, learning objectives, among others |
|  | The elements of the game are created to contemplate different levels of learning |
|  | The game has elements that allow the user to recognize their progress throughout the learning process |
|  | The game has mechanisms to check the player's performance |
| Content | The educational content intended for the game is correctly represented by its elements (consistent, unambiguous, and complete), as well as by the game's execution flow |
|  | The educational content is partitioned into topics and / or subtopics in the game, through its elements |
| Educational agent | The educational agent provides feedback to the player |
|  | The player perceives feelings through pictures, actions or names of the educational agent |
|  | The player always receives polite and expressive feedbacks |

Azevêdo M, Rousy D, Siebra C. AHJED - Avaliação Heurística para Jogos Educacionais Digitais. Nuevas Ideas en Informática Educ 2018;14:126–136.
