# Supplementary material for: A Serious Game (Immunitates) About Immunization: Development and Validation Study
Source: JMIR Serious Games. 2022 Feb 18;10(1):e30738. doi: 10.2196/30738 (PMC8900905; doi:10.2196/30738)
Supplement: Multimedia Appendix 2 [file games_v10i1e30738_app2.docx]

Multimedia Appendix 2

Instrument for content validation by Teixeira et al.

|  | Item |
| --- | --- |
| Group 1 – Objectives | The information/content is consistent with the daily needs of the target audience of the game |
|  | The information/content is important for the quality of the work of the target audience of the game |
|  | Invites and / or instigates changes in behavior and attitude |
|  | It can circulate in the scientific area |
|  | Meets the objectives of institutions in which the target audience of the game work |
| Group 2 – Structure and presentation | The game is appropriate for the target audience |
|  | The messages are presented in a clear and objective way |
|  | The information presented is scientifically correct |
|  | The material is appropriate to the sociocultural level of the target audience of the game |
|  | The information is well structured in agreement and spelling |
|  | The writing style corresponds to the level of knowledge of the target audience |
|  | The information on each navigation button in the game is consistent |
|  | The size of the game title and questions is adequate |
|  | The illustrations are expressive and sufficient |
|  | The material (smartphone game) is appropriate |
|  | The number of questions is adequate |
| Group 3 – Relevance | The themes portray key aspects that must be reinforced |
|  | The game allows the transfer and generalization of learning to different contexts |
|  | The game proposes the construction of knowledge |
|  | The game covers the subjects necessary for the target audience's know-how |
|  | It is suitable for use by the target audience of the game |

Teixeira E, Martins TDR, Miranda PO, Cabral BG, Costa e Silva BA, Rodrigues LSS. Educational technology on postpartum care: development and validation. Rev Baiana Enfermagem‏ [Internet] 2016;30(2):1–10. [doi: 10.18471/rbe.v30i2.15358]
