# Supplementary material for: A Serious Game (Immunitates) About Immunization: Development and Validation Study
Source: JMIR Serious Games. 2022 Feb 18;10(1):e30738. doi: 10.2196/30738 (PMC8900905; doi:10.2196/30738)
Supplement: Multimedia Appendix 3 [file games_v10i1e30738_app3.docx]

Multimedia Appendix 3

Heuristic validation (AHJED instrument) with experts (n=49) and nursing students (n=15). CVI: Content validity index.

Table H1. Interface validation by experts and nursing students. Brasília, Brazil, 2021.

| Heuristics | Experts | | Students | | | |
| --- | --- | --- | --- | --- | --- | --- |
|  | Cronbach alpha | CVI | | Cronbach alpha | CVI |  |
| The user knows their location and easily visualizes their status in the game | 0.87 | 0.94 | | 0.88 | 1 |  |
| The user has free control over their actions in the game environment | 0.87 | 0.92 | | 0.87 | 0.86 |  |
| The elements in the game are consistent and standardized | 0.87 | 0.94 | | 0.88 | 1 |  |
| The elements of the game can prevent the user from accidentally performing an action | 0.87 | 0.84 | | 0.88 | 0.93 |  |
| The elements of the game are suggestive enough to allow the user to play without having to resort to manuals and other types of help | 0.87 | 0.86 | | 0.9 | 0.8 |  |
| The elements of the game allow the user to perform their tasks efficiently, that is, with as little effort as possible | 0.87 | 0.98 | | 0.88 | 1 |  |
| The number of elements in the game is sufficient for the user to achieve their goals without confusing them | 0.87 | 0.92 | | 0.88 | 1 |  |
| The game has elements that help the user to recognize, diagnose, and recover from the mistakes made | 0.87 | 0.82 | | 0.87 | 0.86 |  |
| Total | 0.87 | 0.9 | | 0.88 | 0.93 |  |

Table H2. Playability validation by experts and nursing students. Brasília, Brazil, 2021.

| Heuristics | Experts | | Students | | | |
| --- | --- | --- | --- | --- | --- | --- |
|  | Cronbach alpha | CVI | | Cronbach alpha | CVI |  |
| The game provides enough information for the user to start playing | 0.87 | 0.96 | | 0.87 | 0.93 |  |
| The game's control keys follow conventional standards | 0.87 | 0.88 | | 0.88 | 1 |  |
| Users can save games in different states throughout the game | 0.87 | 0.86 | | 0.88 | 1 |  |
| Users who have successfully completed a game stage are rewarded | 0.87 | 0.92 | | 0.88 | 1 |  |
| Game elements, for example, challenges, should not frustrate game users | 0.86 | 0.84 | | 0.87 | 0.86 |  |
| The elements of the game give the user a sense of immersion, that is, it allows them to feel part of the environment, being able to identify and interact with objects in the scene | 0.86 | 0.84 | | 0.87 | 0.86 |  |
| Total | 0.87 | 0.88 | | 0.88 | 0.94 |  |

Table H3. Multimedia validation by experts and nursing students. Brasília, Brazil, 2021.

| Heuristics | Experts | | Students | | | |
| --- | --- | --- | --- | --- | --- | --- |
|  | Cronbach alpha | CVI | | Cronbach alpha | CVI |  |
| The user must be able to recognize in the multimedia elements of the game, what are the objectives contemplated by them | 0.87 | 0.82 | | 0.87 | 0.93 |  |
| There is a correspondence between the multimedia elements used in the game and the learning contents contemplated in it | 0.87 | 0.84 | | 0.88 | 1 |  |
| The combinations between the multimedia elements used in the game and presented to the user are consistent and representative | 0.87 | 0.9 | | 0.88 | 1 |  |
| The quality of the multimedia elements used is sufficient for the user to understand their purpose (objective) | 0.87 | 0.92 | | 0.88 | 1 |  |
| The multimedia elements of the game contribute to the presentation of the learning content, making it more attractive | 0.86 | 0.86 | | 0.88 | 0.93 |  |
| Total | 0.87 | 0.87 | | 0.88 | 0.97 |  |

Table H4. Artificial intelligence validation by experts and nursing students. Brasília, Brazil, 2021.

| Heuristics | Experts | | Students | | | |
| --- | --- | --- | --- | --- | --- | --- |
|  | Cronbach alpha | CVI | | Cronbach alpha | CVI |  |
| The game's artificial intelligence is consistent | 0.87 | 0.96 | | 0.88 | 0.93 |  |
| The game's artificial intelligence is balanced with the player's skill | 0.87 | 0.92 | | 0.88 | 0.8 |  |
| The game’s artificial intelligence has no flaws or inconsistencies | 0.86 | 0.82 | | 0.87 | 0.93 |  |
| Total | 0.87 | 0.9 | | 0.88 | 0.89 |  |

Table H5. Game’s story validation by experts and nursing students. Brasília, Brazil, 2021.

| Heuristics | Experts | | Students | | | |
| --- | --- | --- | --- | --- | --- | --- |
|  | Cronbach alpha | CVI | | Cronbach alpha | CVI |  |
| The player understands the story of the game clearly and consistently | 0.87 | 0.92 | | 0.88 | 1 |  |
| The story promotes immersion | 0.86 | 0.9 | | 0.87 | 1 |  |
| The game transports the player to a level of emotional involvement. The player is interested in the characters, because (1) they are like me; (2) they are interesting to me, (3) the characters develop as the game progresses | 0.86 | 0.88 | | 0.88 | 0.8 |  |
| Total | 0.86 | 0.9 | | 0.88 | 0.93 |  |

Table H6. Educational elements validation by experts and nursing students. Brasília, Brazil, 2021.

| Heuristics | Experts | | Students | | | |
| --- | --- | --- | --- | --- | --- | --- |
|  | Cronbach alpha | CVI | | Cronbach alpha | CVI |  |
| The user must be able to recognize in the elements of the game, what are the learning objectives contemplated by them | 0.86 | 0.92 | | 0.88 | 1 |  |
| The game should allow its users to have greater autonomy of the learning process, (re) configuring their activities, learning objectives, among others | 0.86 | 0.9 | | 0.88 | 0.93 |  |
| The elements of the game are created to contemplate different levels of learning | 0.86 | 0.86 | | 0.87 | 0.73 |  |
| The game has elements that allow the user to recognize their progress throughout the learning process | 0.87 | 1 | | 0.88 | 1 |  |
| The game has mechanisms to check the player's performance | 0.87 | 0.82 | | 0.87 | 0.67 |  |
| Total | 0.86 | 0.9 | | 0.88 | 0.87 |  |

Table H7. Contents validation by experts and nursing students. Brasília, Brazil, 2021.

| Heuristics | Experts | | Students | | | |
| --- | --- | --- | --- | --- | --- | --- |
|  | Cronbach alpha | CVI | | Cronbach alpha | CVI |  |
| The educational content intended for the game is correctly represented by its elements (consistent, unambiguous, and complete), as well as by the game's execution flow | 0.87 | 0.82 | | 0.88 | 0.86 |  |
| The educational content is partitioned into topics and / or subtopics in the game, through its elements | 0.87 | 0.84 | | 0.88 | 0.67 |  |
| Total | 0.87 | 0.83 | | 0.88 | 0.77 |  |

Table H8. Educational agent validation by experts and nursing students. Brasília, Brazil, 2021.

| Heuristics | Experts | | Students | | | |
| --- | --- | --- | --- | --- | --- | --- |
|  | Cronbach alpha | CVI | | Cronbach alpha | CVI |  |
| The educational agent provides feedback to the player | 0.87 | 0.87 | | 0.87 | 0.8 |  |
| The player perceives feelings through pictures, actions or names of the educational agent | 0.87 | 0.75 | | 0.87 | 0.67 |  |
| The player always receives polite and expressive feedbacks | 0.87 | 0.97 | | 0.87 | 0.8 |  |
| Total | 0.87 | 0.86 | | 0.87 | 0.76 |  |
