# Supplementary material for: A Serious Game (Immunitates) About Immunization: Development and Validation Study
Source: JMIR Serious Games. 2022 Feb 18;10(1):e30738. doi: 10.2196/30738 (PMC8900905; doi:10.2196/30738)
Supplement: Multimedia Appendix 4 [file games_v10i1e30738_app4.docx]

Multimedia Appendix 4

Content validation with the instrument by Teixeira et al. with experts only (n=49). CVI: Content Validity Index.

Table C1. Objectives of the content validation. Brasília, Brazil, 2021.

| Item | Cronbach alpha | CVI |
| --- | --- | --- |
| The information/content is consistent with the daily needs of the target audience of the game | 0.9 | 0.88 |
| The information/content is important for the quality of the work of the target audience of the game | 0.9 | 1 |
| Invites and / or instigates changes in behavior and attitude | 0.89 | 0.73 |
| It can circulate in the scientific area | 0.9 | 0.88 |
| Meets the objectives of institutions in which the target audience of the game work | 0.89 | 0.92 |
| Total | 0.9 | 0.88 |

Table C2. Structure and presentation of the content validation. Brasília, Brazil, 2021.

| Item | Cronbach alpha | CVI |
| --- | --- | --- |
| The game is appropriate for the target audience | 0.9 | 0.92 |
| The messages are presented in a clear and objective way | 0.9 | 0.78 |
| The information presented is scientifically correct | 0.9 | 0.9 |
| The material is appropriate to the sociocultural level of the target audience of the game | 0.89 | 0.9 |
| The information is well structured in agreement and spelling | 0.9 | 0.98 |
| The writing style corresponds to the level of knowledge of the target audience | 0.9 | 0.94 |
| The information on each navigation button in the game is consistent | 0.9 | 0.88 |
| The size of the game title and questions is adequate | 0.9 | 0.86 |
| The illustrations are expressive and sufficient | 0.9 | 0.82 |
| The material (smartphone game) is appropriate | 0.89 | 0.96 |
| The number of questions is adequate | 0.9 | 0.96 |
| Total | 0.9 | 0.9 |

Table C3. Relevance of the content validation. Brasília, Brazil, 2021.

| Item | Cronbach alpha | CVI |
| --- | --- | --- |
| The themes portray key aspects that must be reinforced | 0.9 | 0.94 |
| The game allows the transfer and generalization of learning to different contexts | 0.89 | 0.92 |
| The game proposes the construction of knowledge | 0.89 | 0.9 |
| The game covers the subjects necessary for the target audience's know-how | 0.89 | 0.94 |
| It is suitable for use by the target audience of the game | 0.89 | 0.94 |
| Total | 0.89 | 0.93 |
